# Plasma Apolipoproteins Predicting the Occurrence and Severity of Diabetic Retinopathy in Patients With Type 2 Diabetes Mellitus

**DOI:** 10.3389/fendo.2022.915575

**Published:** 2022-07-22

**Authors:** Xinyuan Zhang, Yao Nie, Zhizhong Gong, Meidong Zhu, Bingjie Qiu, Qiyun Wang

**Affiliations:** ^1^ Beijing Tongren Eye Center, Beijing Tongren Hospital, Capital Medical University, Beijing, China; ^2^ Beijing Retinal and Choroidal Vascular Disorders Study Group, Beijing, China; ^3^ Division of Medical Affairs, Beijing Hospital of Traditional Chinese Medicine, Capital Medical University, Beijing, China; ^4^ New South Wales Tissue Bank, New South Wales Organ and Tissue Donation Service, Sydney, NSW, Australia; ^5^ Save Sight Institute, Discipline of Clinical Ophthalmology and Eye Health, University of Sydney, Sydney, NSW, Australia

**Keywords:** dyslipidemia, apolipoprotein profiles, diabetes mellitus, diabetic retinopathy, biomarkers

## Abstract

**Objective:**

Apolipoproteins are amphipathic molecules and the major components of plasma lipoproteins. This study aims to investigate the effects of dysregulated apolipoprotein (apo) profiles and their ratios on type 2 diabetes mellitus (T2DM) and diabetic retinopathy (DR) further to test the hypothesis that altered serum level of apolipoproteins is strong biomarkers for DR.

**Research Design and Methods:**

This case-control study consists of 157 patients with T2DM including DM without DR, non-proliferative DR (NPDR), and proliferative DR (PDR). Fifty-eight age- and sex-matched healthy subjects were enrolled as normal controls. Blood biochemistry profile including serum levels of glucose, glycated hemoglobin (HbA1c), lipid profile [total cholesterol (TC), Triglycerides (TG), high and low-density lipoprotein (HDL-C and LDL-C)] was estimated. Apolipoproteins (apos, A-I, A-II, B, C-II, C-III, and E) was evaluated by protein chips (Luminex technology). Apolipoprotein ratios and arteriosclerosis-associated plasma indices were calculated. The Kruskal–Wallis test, independent sample t-test or Mann–Whitney U test, and multivariate regression analysis were performed to investigate the association of serum lipid biomarkers and the DR severity.

**Results:**

Serum level of apoA-I was negatively correlated with TC-(HDL-C)/HDL-C (*p* < 0.001), fasting glucose (*p* < 0.001), HbA1c (*p* < 0.001), and (*p*<0.001), while apoE, apoC-II/apoC-III, apoA-II/apoA-I were positively correlated with above traditional biomarkers (*p* < 0.001). Single variable logistic analysis results showed that body mass index (BMI) (*p* = 0.023), DM duration (*p* < 0.001), apoE (*p* < 0.001), apoC-II/apo C-III (*p* < 0.001), apoE/apoC-II (*p* < 0.001), atherogenic index (*p* = 0.013), fasting glucose (*p* < 0.001), HbA1c (*p* < 0.001), LPA (*p* = 0.001), and LDL-C/HDL-C (*p* = 0.031) were risk factors for the occurrence and severity of DR. Multivariate logistic regression mode showed that apoC-II/apoC-III and apoB/non–HDL-C (*p* < 0.001) as well as apoE/apoC-II (*p* = 0.001) were the independent risk factors for the occurrence and severity of DR—apopA-I and apoA-II are protective factors for DR—after controlling for the duration of DM, HbA1c, fasting glucose, and LPA.

**Conclusions:**

apoE, apoC-II/apoC-III, apoE/apoC-II, and apoB/non–HDL-C could be used as novel biomarkers for occurrence and severity of DR, whereas apoA-I and apoA-II resulted as protective factors for DR.

## Introduction

Diabetic retinopathy (DR) is the leading cause of blindness among the working-age populations (1, 2). According to the International Diabetes Federation, the global prevalence of diabetes in 2019 is 9.3% (463 million people), rising to 10.9% (700 million) by 2045. A meta-analysis, which included 35 cohort studies, indicated that the global prevalence of any DR is 34.6%, vision-threatening DR is 10.2%, accounting for 51% of blindness cases worldwide (3). Furthermore, Diabetes Control and Complications Trial (DCCT)/Epidemiology of Diabetes Interventions and Complications (EDIC) (4) and Steno-2 (5) studies showed that about 53% and 51% enrolled subjects with type 2 diabetes mellitus (T2DM) still developed DR after 6.5–13.3 years, respectively, regardless of strict interventions of hyperglycemia, blood pressure, and lowering lipids they underwent. This further indicated that standard intervention could partially reduce the risk of DR but cannot prevent the development of DR.

Although traditional plasma lipid profiles have been implicated in the pathogenesis of DR, existing studies did not provide consistent results, and a conclusive link remains elusive. Some studies have not reported any effects of lipids in the pathogenesis of DR. For example, the Wisconsin Epidemiologic Study of Diabetic Retinopathy (WEDRS study) did not find the correlation between the plasma level of total cholesterol (TC) and HDL-C with DR severity and retinal hard exudates (6). DCCT/ECCT also reported that TC, triglycerides, HDL-C, and non–HDL-C cholesterol were not associated with DR severity (7). Treatment with stains did not show any significant benefit for DR progression (8, 9). Furthermore, the Fenofibrate Intervention and Event Lowering in Diabetes (FIELD) (10) study and the Action to Control Cardiovascular Risk in Diabetes (ACCORD) Eye Study (11) did not show the causal associations of fenofibrate with the lipid profiles in preventing the progression of DR, indicating that the beneficial effects of fenofibrate on DR are not correlated with changes of plasma lipid levels but may exert their effect through modulating the expression level of apolipoproteins (12).

Apolipoproteins are amphipathic molecules and certain multifunctional proteins. As an important component of lipoproteins, they regulate the transport and distribution of lipoproteins, promote binding of lipoproteins to cell surface receptors, enhance lipid uptake capacity of cells, and activate lipid enzymes (13). Apolipoproteins are associated with a variety of diseases, including diabetic macro- and microvasculopathy (14). Dysregulated apolipoproteins A and B have been implicated in DR (15); yet, the role of apoC-II, apoC-III, apoE, and other family members of apolipoproteins in the pathogenesis of DR is still uncertain and has not been reported.

We have previously shown that arteriosclerosis-associated plasma parameters, atherogenic plasma index (API; LDL-C/HDL-C), atherogenic index (AI; TC-(HDL-C)/HDL-C), atherogenic index of plasma [AIP; Log (TG/HDL-C)] are novel biomarkers and more sensors for the occurrence and progression of DR (16), which provided new insights into the treatment of DR. In comparison with the traditional lipid profiles, a number of studies have confirmed that these indices are more sensitive in predicting the progression of diabetes and DR. Nonetheless, the synergetic effects of apolipoproteins profiles with dyslipidemia, hemoglobin, plasma glucose, and the atherogenic indices need to be further studied.

To address these gaps in the literature, we examined the relationship of plasma level of apolipoproteins profiles including apoA-I, apoA-II, apoB, apoC-II, apoC-III, and apoE, and the ratios of apoB/apoA-I, apoB/Non–HDL-C, apoC-II/apoC-III, apoA-II/apoA-I, and apoE/apoC-II *with* type 2 diabetes (no DR) and DR. We further tested the hypothesis that altered plasma levels of apolipoproteins are biomarkers in T2DM and DR.

## Materials and Methods

### Participants’ Characteristics

The study per the tenets set forth in the Declaration of Helsinki. All the participants signed an informed consent which was approved by the Ethics Committee of Beijing Tongren Hospital, Capital Medical University before enrollment.

This case-control study consecutively recruited 157 Han Chinese patients with T2DM and 58 age- and sex-matched normal without DM controls at Beijing Tongren Hospital between September 2016 and September 2020. Type 2 diabetes and DR were defined according to the 2020 American Diabetes Association (ADA) guideline and 2017 A Position Statement of DR, respectively. Participants without DR in either eye were assigned to the “DM” group, whereas those with DR were categorized as non-proliferative DR (NPDR) group and those with retinal and/or optic disc neovascularization in at least one eye as PDR group.

The inclusion criteria were described previously (16). The exclusion criteria were the following: coexistence of other retinal diseases including retinal vein occlusion, macular hole, macular pucker, age-related macular degeneration, and inherited retinal diseases; recent history of eye anterior or posterior segment surgery; ocular media opacity; and inability to tolerate examinations due to severe system diseases; patients on lipid-lowering therapy or with history of lipid disorders were also excluded (16). Subjects with cardiovascular diseases including angina and stroke as well as other server system disorders were also excluded.

### Comprehensive Eye Examination

Best-corrected visual acuity (BCVA), non-contact intraocular pressure (TX20 Automatic Non-contact Tonometer, Canon Co., Ltd, Tokyo, Japan), slit-lamp microscopic examination (SL-IE Slit Lamp Microscope, Topcon Co., Ltd, Tokyo, Japan), and fundus photography (CR-1 non-mydriatic Fundus Camera, Canon Co., Ltd) with mydriasis and capture of least two-field centered on optic disc and macula of both eyes were tested in all the participants. All subjects were examined by SS-OCT (DRI OCT1 Atlantis scanner, Topcon Co., Ltd., Tokyo, Japan or Plex Elite 9000, Carl Zeiss Meditec, Inc, Oberkochen, German). A 9 mm × 9 mm scanning range mode was selected to obtain B-scan images. The individual’s DR grade was recorded as the degree of worse eye.

### Criteria of Dyslipidemia

Fasting blood samples were obtained from each participant within 2 weeks of eye examinations; routine plasma biochemistry test included fasting glucose, glycated hemoglobin (HbA1c), TC, and LDL cholesterol (LDL-C), high-density lipoprotein cholesterol (HDL-C), triglycerides, lipoprotein (a). Criteria of hyperlipidemia bases on the National Cholesterol Education Program (NCEP) Adult Treatment Panel-III (ATP-III) report in 2001 and the guidelines for the prevention and treatment of dyslipidemia in Chinese adults 2016. Cholesterol (total cholesterol), (TC) >5.17 mmol/L, triglyceride (TG) >1.70 mmol/L, or LDL-C > 3.37 mmol/L was defined hyperlipidemia.

All participants underwent a standardized assessment of other risk factors, including age, gender, and duration of diabetes, duration of hypertension (HBP, high blood pressure), and body mass index (BMI).

### Determination of the Cutoff Value of apoE/apoC-II, apoC-II/apoC-III, apoB/non–HDL-C, apoA-II/apoA-I, apoB/apoA-I, apoB/apoA-II, apoB/non–HDL-C, API, and AI by Receiver Operating Characteristic Curve

The indices including apoE/apoC-II, apoC-II/ApoC-III, apoB/non-HDL, apoA-II/apoA-I, apoB/apoA-I, apoB/apoA-II, API, and AI were estimated by receiver operating characteristic (ROC) curve. The indices with high sensitivity and specificity were selected as the cutoff values on the ROC curve. apoE/apoC-II 0.22 [receiver operating characteristic curve (AUC): 0.74, sensitivity = 0.55, specificity = 0.85], apoC-II/apoC-III 0.52 (AUC:0.70, sensitivity = 0.60, specificity = 0.71), apoB/non–HDL-C 0.22 (AUC: 0.74, sensitivity = 0.97, specificity = 0.46), apoA-II/apoA-I 0.67 (AUC: 0.60, sensitivity = 0.58, specificity = 0.66), were determined as the cut-off value in this study. apoB/apoA-I 2.7 (AUC: 0.60, sensitivity = 0.50, specificity = 0.6). API 2.24 (AUC: 0.75; sensitivity = 0.71, specificity = 0.52), AI 2.91 (AUC, 0.72; sensitivity = 0.63; specificity = 0.72), AIP (AUC 0.564; sensitivity = 0.607, specificity = 0.552) were selected as the cutoff value as we described previously (16).

### Determination of the Plasma Level of Apolipoproteins

Plasma level of apolipoproteins (A-I, A-II, B, C-II, C-III, and E) were determined by Luminex technology (Luminex 200™ liquid chip detector, Millipore, Boston, Massachusetts, USA) according to the manufacturer’s instructions.

### Sample Size Calculation

Based on a pilot study to detect the difference of the serum level of lipid and lipoproteins profiles as we described (17), the sample size was calculated with the designed power (1-beta = 90%) at a 95% confidence level (alpha = 0.05) using the Power Analysis and Sample Size software (PASS 2022, NCSS LLC, Utah, USA). We determined that the minimum sample size was 25 subjects in the study and control groups (per arm) to detect the difference between means of the expression level of apos and lipid profiles which had diagnostic performance determined by ROC as we described above. Considering the variability in the plasma level of lipid and apolipoprotein profiles, highly variable parameters can be found even in a healthy population, the sample size was increased to >40 in the present study.

### Statistical Analysis

SPSS software (SPSS, Inc. 23.0, Chicago, IL, USA) was used for the statistical analysis. Baseline parameters including the age of participants, duration of diabetes and HBP, BMI, fasting blood glucose, HbA1c, lipid profiles, and apolipoprotein profiles were described by means ± standard deviation (means ± SD) or median (interquartile range). Normality was assessed by the Kolmogorov–Smirnov test and the Shapiro–Wilk test. The Levene’s test was applied to test the homogeneity of variance. According to data distribution, the comparisons among groups were analyzed by one-way analysis of variance (ANOVA) or the Kruskal–Wallis test. Single variable logistic regression was performed to assess the association between the plasma levels of lipids, apolipoproteins, and DR severity. Multiple and ordered logistic regression models were utilized to assess associations between plasma lipids or apolipoproteins and DR severity categories (no DM, DM with no DR, and NPDR, and PDR). The association between the levels of apolipoproteins, the indices, and the traditional biochemical marks was assessed by Spearman’s rank correlation coefficient. *P*-value < 0.05 was statistically significant.

## Results

### Baseline Demographic and Clinical Characteristics

We recruited 215 subjects (121 males and 94 females, aged 27–82 years old), including 157 patients with type 2 diabetes, from the outpatient clinic of Beijing Tongren Hospital. The participants were assigned to the DM group if they had no DR, NPDR, and PDR. Fifty-eight age- and sex-matched normal without DM subjects were enrolled as the normal control group (aged 30–74 years) (**Table 1**). Duration of DM was defined as from first diagnosis with an International Classification of Disease code until the time of recruitment as DR or DM in this study.

**Table 1:** Demographic characteristics of the enrolled subjects and the biochemical parameters including lipid profiles and arteriosclerosis-associated plasma indices in subjects with normal, DM, NPDP, and PDR.

Group	Normal without DM	DM	NPDR	PDR	H/x^2^/F	*P* Value
Number	58	44	59	54	–	–
Age, years	58.00 (46.50–64.50)	57.00 (49.00–65.00)	56.00 (49.25–61.00)	53.00 (46.25–60.75)	5.5	0.139
Male sex, n (%)	26 (45)	25 (57)	38 (64)	32 (59)	4.88	0.181
Duration of DM, years	0	10.00 (5.00–15.00)	10.00 (6.25–16.75)	14.00 (10.00–17.00)	127.95^b**^	<0.001^**^
Duration of HBP, years	0.00 (0.00–0.00)	2.00 (0.00–10.00)	0.00 (0.00–6.00)	1.00 (0.00–5.00)	27.25^b**^	<0.001^**^
BMI, kg/m^2^	23.96 (22.51–25.66)	25.77 (23.51–28.22)	25.35 (23.41–27.68)	24.71 (22.87–27.62)	7.53^b^	0.057
Fasting blood glucose, mmol/L	5.40 (4.98–5.89)	7.49 (6.17–8.79)	8.05 (6.68–10.23)	8.34 (6.83–10.50)	82.17^b**^	<0.001^**^
HbA1c, mmol/mol	37.71 (35.52–40.98)	50.82 (45.36–59.56)	62.84 (51.91–72.68)	65.03 (53.01–79.24)	108.61^b**^	<0.001^**^
HbA1c, %	5.60 (5.40–5.90)	6.80 (6.20–7.60)	7.90 (6.85–8.85)	8.10 (6.98–9.40)	108.61^b**^	<0.001^**^
Triacylglycerol, mmol/L	1.28 (0.94–1.65)	1.63 (1.00–2.53)	1.20 (0.86–2.13)	1.43 (1.02–2.75)	4.92^b^	0.178
Cholesterol, mmol/L	4.93 (4.53–5.77)	4.29 (3.70–5.05)	4.31 (3.85–5.34)	5.01 (4.36–5.89)	19.36^b**^	<0.001^**^
LDL-C, mmol/L	3.03 ± 0.11	2.48 ± 0.14	2.77 ± 0.12	3.08 ± 0.10	5.04^a*^	0.002^*^
HDL-C, mmol/L	1.35 (1.12–1.63)	1.19 (0.95–1.40)	1.15 (0.99–1.47)	1.15 (0.98–1.43)	11.64^b*^	0.009^*^
LPA	10.20 (4.83–23.50)	8.70 (3.15–26.78)	11.10 (6.60–30.70)	20.45 (7.60–57.20)	10.18^b*^	0.017^*^
API	2.30 ± 0.11	2.21 ± 0.14	2.32 ± 0.107	2.64 ± 0.11	2.64^a^	0.05
AI	2.71 (1.88–3.75)	2.75 (2.07–3.93)	2.71 (2.23–3.68)	3.19 (2.52–3.85)	6.48^b^	0.09

There were significant differences in the duration of DM and HBP, fasting blood glucose, HbA1c, TC, LDL-C, HDL-C, and LPA among the four groups.*Statistically significant: p ≤ 0.05. **Statistically significant: p ≤ 0.001. According to the type of data and the data distribution, one-way ANOVA analysis (a), post-hoc LSD correction, and Kruskal–Wallis (b) analysis were applied. DM, diabetes mellitus; apo, apolipoprotein. HbA1c, glycated hemoglobin, HDL-C, high-density lipoprotein cholesterol. LDL-C, low-density lipoprotein cholesterol; API/AI, atherogenic index of plasma; API, atherogenic plasma index: LDL-C/HDL-C; AI, atherogenic index: TC- (LDL-C)/HDL-C; BMI, body mass index; TC, total cholesterol; TG, triglyceride; LPA, lipoprotein (a).

There were no statistically significant differences in age or gender between the four groups (*p_age_* = 0.139, *p_gender_* = 0.181). The duration of DM was shorter in the normal without DM group compared to NPDR and PDR groups (*p_DM_* vs.*p_Normal_ <* 0.001,*p_NPDR_* vs.*p_Normal_ <* 0.001, *p_PDR_* vs.*p_Normal_ <* 0.001). Fasting blood glucose in the normal without DM group was significantly lower than that in the DM, NPDR, and PDR (*p_DM_* vs. *p_Normal_ <* 0.001,*p_NPDR_* vs.*p_Normal_ <* 0.001, *p_PDR_* vs.*p_Normal_ <* 0.001). There was a significant difference in glycosylated HbAc1 levels between the four groups (*p_DM_* vs.*p_Normal_ <* 0.001,*p_NPDR_* vs. *p_normal_ <* 0.001, *p_PDR_* vs.*p_Normal_ <* 0.001, *p_PDR_* vs. *p_DM_ =* 0.006). Significant differences in the level of TC, LDL-C, HDL-C, and LPA were found between the four groups (*p_TC_* < 0.001, *p_LDL-C_* = 0.002, *p_HDL-C_* = 0.009, and *p _LPA_* = 0.017) (**Table 1**).

### Correlations Between the Levels of Apolipoproteins and the Traditional Plasma Lipid Biochemical Markers

**Figure 1:**
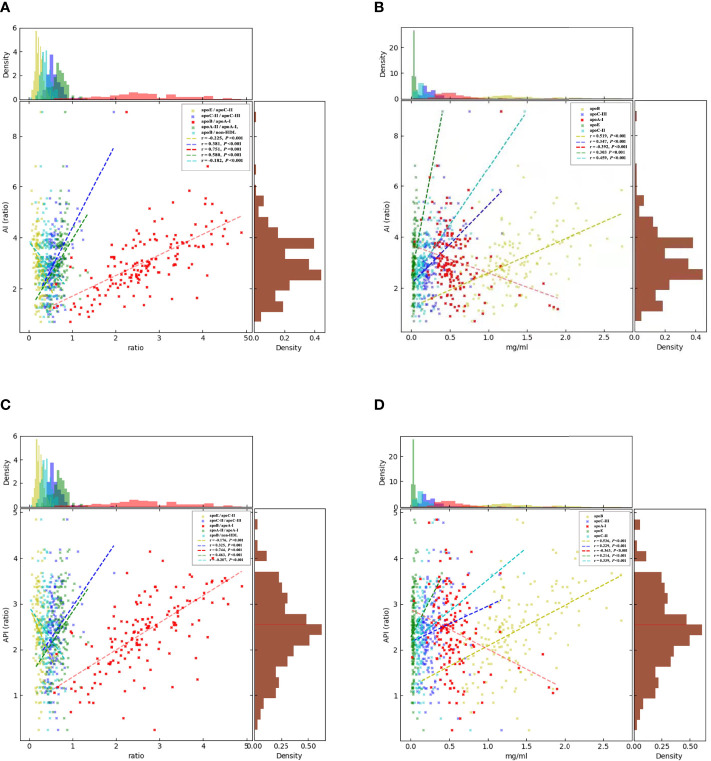
Correlations between the circulating levels of AI, API, and the apolipoproteins by Spearman’s rank correlation coefficient analysis. **(A)**: apoB/apoA-I (*p* < 0.001), apoC-II/apoC-III (*p* < 0.001), and apoA-II/apoA-I (*p* < 0.001) were positively correlated with AI. apoB/non–HDL-C (*p* < 0.001) and apoE/apoC-II were significantly negatively corelated with AI. **(B)**: apoC-III (*p* < 0.001), apoE (*p* < 0.001), apoC-II, and apoB were positively correlated with AI. **(C)**: apoB/apoA-I (*p* < 0.001), apoC-II/apoC-III (*p* < 0.001), and apoA-II/apoA-I (*p* < 0.001) were significantly positively correlated with API. apoB/non–HDL-C (*p* < 0.001) and apoE/apoC-II were significantly negatively correlated with API. **(D)**: apoA1 (*p* < 0.001), apoC-III (*p* < 0.001), apoE (*p* < 0.001), apoC-II, and apoB were positively correlated with API, but apoA-I was significantly negatively correlated with API. The brown histograms on the right side represent the data distribution of AI and API. AI, atherogenic index was calculated as TC-(LDL-C)/HDL-C. API, atherogenic plasma index was defined as LDL-C/HDL-C. apo, apolipoprotein.

**Figure 2:**
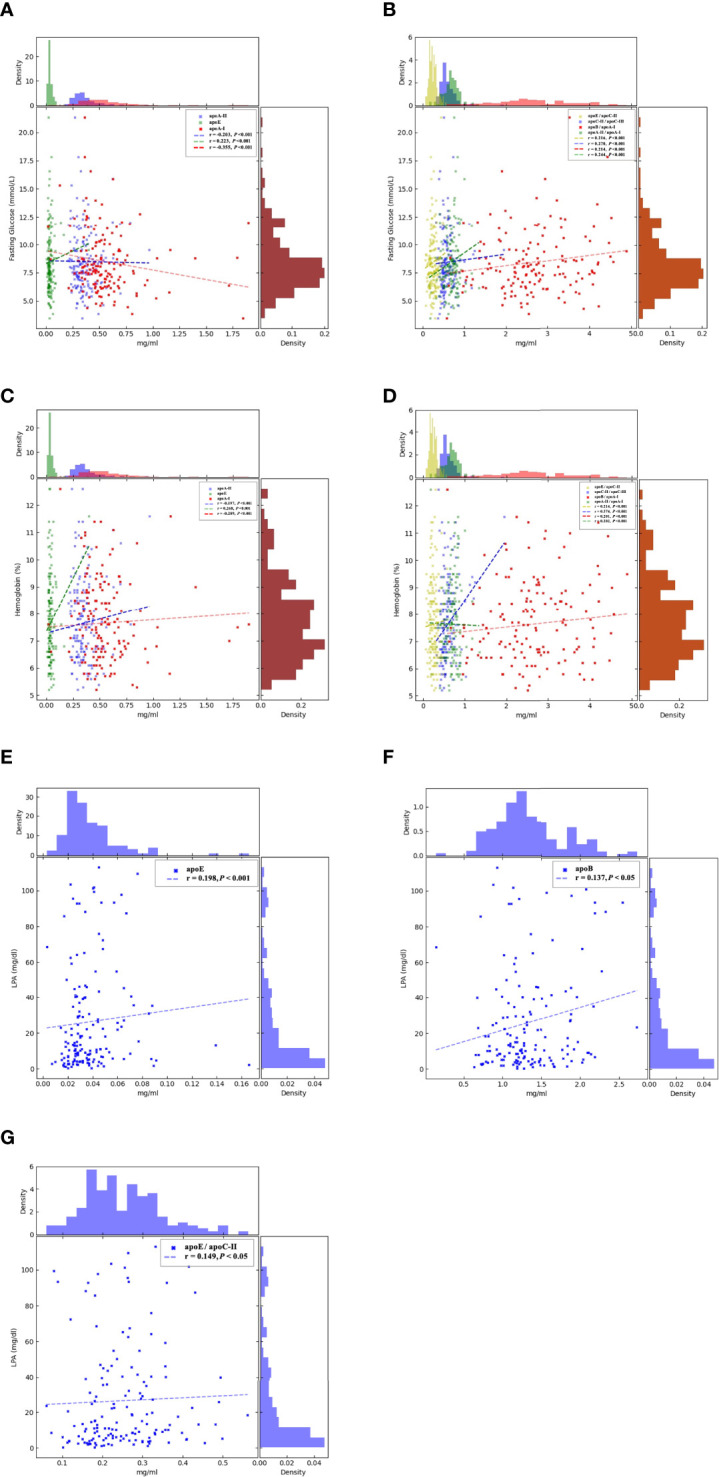
Correlations between the circulating levels of fasting glucose, hemoglobin, LPA, and the apolipoproteins by Spearman’s rank correlation coefficient analysis. **(A)**: apoA-I (*p* < 0.001) and apoA-II (*p* < 0.001) were significantly negatively correlated with fasting glucose, but apoE (*p* < 0.001) was positively correlated. **(B)**. apoB/apoA-I (*p* < 0.001), apoC-II/apoC-III (*p* < 0.001), apoA-II/apoA-I (*p* < 0.001), and apoE/apoC-II (*p* < 0.001) were significantly positively correlated with fasting glucose. **(C)**. apoA-I (*p* < 0.001) and apoA-II (*p* < 0.001) were significantly negatively correlated with hemoglobin, but apoE (*p* < 0.001) was positively correlated. **(D)**. apoB/apoA-I (*p* < 0.001), apoC-II/apoC-III (*p* < 0.001), apoA-II/apoA-I (*p* < 0.001), and apoE/apoC-II (*p* < 0.001) were significantly positively correlated with hemoglobin. The brown histograms on the right side represent the data distribution of fasting blood glucose (mmol/L) and hemoglobin (%). AI, atherogenic index was calculated as TC-(LDL-C)/HDL-C. API, atherogenic plasma c LDL-C/HDL-C. apo, apolipoprotein. apoE, apoB, and apoE/apoC-II were positively corrected with LPA, apoE (*p* < 0.001) **(E)**, apoB (*p* < 0.05) **(F)**, and apoE/apoC-II (*p* < 0.05) **(G)**. LPA, Lipoprotein(a); apo, apolipoprotein.

Spearman’s rank correlation coefficient analysis was utilized to determine the effects of apolipoproteins on the traditional lipid biomarkers in the occurrence and severity of DR. apoA-I was significantly negatively correlated with AI (r = −0.39, *p*_AI_ < 0.001)(**Figure 1**), fasting glucose(r _=_ −0.36, *p* < 0.001), HbA1c (r = −0.29, *p* < 0.001) (**Figure 2**), and API (r _=_ −0.36, *p* < 0.001) (**Figure 1**); apoA-II was significantly negatively correlated with fasting glucose (r = −0.20 *P* < 0.001), HbA1c (r = −0.20, *p* < 0.001) (**Figure 2**), apoB/non–HDL-C (r = −0.18, *p* < 0.001), and AIP (r = −0.21, *p* < 0.001 (**Figure 1**). apoE/apoC-II was negatively correlated with AI (r = −0.23, *p* < 0.001) and API (r = −0.18, *p* < 0.001), and apoC-III, apoE, apoB, apoC-II, apoB/apoA-I, apoC-II/apoC-III, and apoA-II/apoA-I were positively correlated with AI (*p* < 0.001) and API (*p* < 0.001), which indicated that they contributed to the occurrence of T2DM and severity of DR.

### Risk and Protective Factors for DR (Comparison Between the No DM, DM With No DR, NPDR, and PDR Groups)

To further evaluate the associations of baseline parameters, traditional biochemical and apolipoprotein profiles in the occurrence, and severity of DR, a single-variable ordered logistic analysis was performed. The results showed that BMI (OR = 1.09, 95% CI 1.01–1.18, *p* = 0.023), DM duration (OR = 1.19, 95%CI 1.14–1.24, *p* < 0.001), fasting glucose (OR = 1.42, 95% CI:1.27–1.58, *p* < 0.001), HbA1c (OR = 2.55, 95% CI: 2.06–3.15, *p* < 0.001), LPA (OR = 1.02, 95%CI: 1.01–1.03, *p* = 0.001), AI (OR = 1.30, 95% CI: 1.06–1.60, *p* = 0.013), and API (OR = 1.03, 95%CI: 1.00–1.06, *p* = 0.031) as well as the plasma levels of apolipoproteins including apoE (OR = 1.30, 95%CI:1.13–1.50, *p* < 0.001), apoC-II/apoC-III (OR = 1.86, 95%CI: 1.53–2.34, *p* < 0.001), apoE/apoC-II (OR = 1.93, 95%CI: 1.47–2.54, *p* < 0.001) were independent risk factors in the occurrence and severity of DR. Moreover, apoA-II (OR = 0.69, 95%CI: 0.54–0.88, *p =* 0.003) and apoB/non–HDL-C (OR = 0.75, 95%CI:0.60–0.94, *p* = 0.011) resulted as independent protective factors in the occurrence of type 2 diabetes, DR, and the severity of DR (**Table 2**).

**Table 2:** Single-variable ordered logistic analysis results showing the associations of baseline parameters, traditional biochemical, and apolipoprotein profiles in the occurrence and severity of diabetic retinopathy (normal without DM, DM, NPDR, and PDR as the dependent variable).

	OR 95% CI	*P* Value
Sex (M vs. F)	1.55 (0.95, 2.51)	0.079
Age per 10years	0.82 (0.65, 1.02)	0.076
BMI, kg/m^2^	1.09 (1.01, 1.18)	0.023*
Duration of DM, years	1.19 (1.14, 1.24)	<0.001**
Duration of HBP, years	1.03 (0.99, 1.07)	0.181
Duration of HBP, years (ref <15 years)	1.06 (0.45, 2.46)	0.897
apoA-I, mg/ml per 0.1	0.92 (0.83, 1.01)	0.082
apoC-III, mg/ml per 0.1	0.87 (0.74, 1.01)	0.071
apoE, mg/ml per 0.01	1.30 (1.13, 1.50)	<0.001**
apoA-II, mg/ml per 0.1	0.69 (0.54, 0.88)	0.003*
apoB, mg/ml per 0.1	0.96 (0.91, 1.01)	0.141
apoC-II, mg/ml per 0.1	1.10 (0.93, 1.30)	0.255
apoB/apoA-I	1.00 (0.95, 1.06)	0.975
apo B/non–HDL-C per 0.1	0.75 (0.60, 0.94)	0.011*
apoC-II/apoC-III per 0.1	1.90 (1.53, 2.34)	<0.001**
apoA-II/apoA-I per 0.1	1.00 (0.98, 1.02)	0.858
apoE/apoCII per 0.1	1.93 (1.47, 2.54)	<0.001**
AI	1.30 (1.06, 1.60)	0.013*
fasting blood glucose, mmol/L	1.42 (1.27, 1.58)	<0.001**
HbA1c, %	2.55 (2.06, 3.15)	<0.001**
LPA, mg/dl	1.02 (1.01, 1.03)	0.001**
API per 0.1	1.03 (1.00, 1.06)	0.031*
TC, mmol/L	1.06 (0.85, 1.32)	0.613
TG, mmol/L	1.15 (0.97, 1.36)	0.115
LDL-C, mmol/L	1.10 (0.83, 1.45)	0.507
HDL-C, mmol/L	0.80 (0.53, 1.18)	0.247

BMI, duration of DM, apoE, apoCII/CIII, apoE/CII, AI, API, fasting blood glucose, and HbA1c were significant independent risk factors for DR severity, but A-II and apoB/non–HDL-C were independent protective factor for DR occurrence and severity.

*Statistically significant: p ≤ 0.05. **Statistically significant: p ≤ 0.001. DM, diabetes mellitus; apo, apolipoprotein; HbA1c, glycated hemoglobin; HDL-C, high-density lipoprotein cholesterol; LDL-C, low-density lipoprotein cholesterol; API/AI, atherogenic index of plasma; API, atherogenic plasma index: defined as LDL-C/HDL-C; AI, atherogenic index: TC- (LDL-C)/HDL-C; BMI, body mass index; TC, total cholesterol; TG, triglyceride; LPA, Lipoprotein (a).

### The Correlations Between the Traditional Risk Factors of DR and Apolipoproteins in the Occurrence and Severity of DR

**Figure 3:**
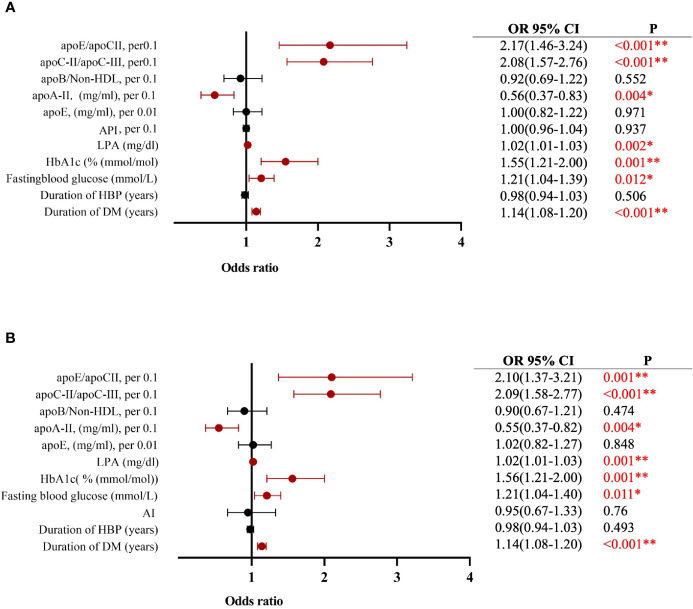
Multiple-variable ordered logistic model showing **(A)** apoC-II/apoC-III and apoE/apoc-II contribute to DM and DR associated with DM duration, fasting glucose, hemoglobin, and lipoprotein a; apoA-II is a protector for DM and DR (when was considered as an independent variable). **(B)** Multiple-variable ordered logistic model showing apoC-II/apoC-III and apoE/apoc-II contribute to DM and DR associated with DM duration, fasting glucose, hemoglobin and lipoprotein (a; apoA-II is a protector for DM and DR when AI was considered as an independent variable). The histograms on the right side represent the data distribution of LPA (mg/dl). *Statistically significant: *p* ≤ 0.05. **Statistically significant: *p* ≤ 0.001. DM, diabetes mellitus; apo, apolipoprotein; HbA1c, glycated hemoglobin, LPA, lipoprotein a. API: LDL-C/HDL-C; AI: (TC-HDL-C)/HDL-C.

Multiple-variable ordered logistic analysis showed that DM duration, fasting glucose, HbA1c, and LPA had a synergetic pathological effect with apoC-II/apoC-III and apoE/apoC-II in the normal without DM, DM, NPDR, and PDR groups. **Table 2** shows that, when DM duration, HBP, fasting blood glucose, HbA1c, LPA, apoE, apoA-II, apoB/non–HDL-C, apoC-II/apoC-III, apoE/apoC-II, and AI were used as the independent variable, DM duration (OR = 1.14, 95%CI 1.08–1.20, *p* < 0.001), fasting glucose (OR = 1.21, 95%CI 1.04–1.40, *p* = 0.011), LPA (OR = 1.02, 95%CI 1.01–1.03, *p* < 0.001), apoC-II/apoC-III, per 0.1 (OR = 2.09, 95%CI 1.58–2.77, *p* < 0.001), and apoE/apoC-II (OR = 2.10, 95%CI 1.37–3.21, *p* = 0.001) contributed to the occurrence and severity of DR, whereas apoA-II (OR = 0.55, 95%CI 0.37–0.82, *p* = 0.004) was a strong protective factor in T2DM and DR (**Figure 3B**).

Similarly, when DM duration, HBP, fasting blood glucose, HbAc1, LPA, apoE, apoA-II, apoB/non–HDL-C, apoC-II/apoC-III, apoE/apoC-II, and API were used as the independent variable, DM duration (OR = 1.14, 95%CI 1.08–1.20, *p* < 0.001), fasting glucose (OR = 1.21, 95%CI 1.04–1.39, *p* = 0.012), LPA (OR = 1.02, 95%CI 1.01–1.03, *p* = 0.002), apoC-II/apoC-III, per 0.1 (OR = 2.08, 95%CI 1.57–2.76, *p* < 0.001), and apoE/apoC-II, per 0.1 (OR = 2.17, 95%CI 1.46–3.24, *p* < 0.001) contributed to the occurrence and severity of DR, whereas apoA-II (OR = 0.56, 95%CI 0.37–0.83, *p* = 0.004) resulted as a protective factor in DR. These results further confirmed that apolipoproteins, especially apoE, apoC-II, and apoC-III, were strong biomarkers for predicting the risk of T2DM and DR (**Figure 3A**).

## Discussion

In this study, we showed that plasma level of apoA-I was significantly negatively correlated with AI, fasting glucose, HbA1c, and API, whereas apoE and apoC-II/apoC-III were significantly positively correlated with fasting glucose and HbA1c, and apoA-II was significantly negatively correlated with fasting glucose and HbA1c. Meanwhile, apoA-II/apoA-I, apoE, apoB, and apoB/apoA-I were significantly positively correlated with the above traditional biomarkers of DR. Single-variable logistic analysis showed that BMI, DM duration, apoE, apoB/non–HDL-C, apoC-II/apoC-III, apoE/apoC-II, AI, fasting glucose, glycated HbA1c, LPA, and API were risk factors of the occurrence and severity of DR. Multivariate ordered logistic regression analysis further confirmed that DM duration, apoC-II/apoC-III, apoE/apoC-II, glycated HbA1c, fasting glucose, and LPA were independent risk factors for the occurrence and severity of DR, whereas apoA-II was a protective factor for DR. To the best of our knowledge, this is the first study that clearly elucidated that apolipoprotein profiles were closely associated with T2DM and DR and could be used as strong plasma biomarkers for predicting the risk of the presence of T2DM and DR as well as severity of DR. apoA-I and apoA-II resulted as protectors for severity of DR.

Apolipoprotein is a group of plasma proteins that binds to lipids to constitute lipoproteins. As an important structural component of lipoproteins and amphipathic molecular, apolipoprotein regulates the transport and distribution of lipoproteins, promote the binding of lipoproteins to cell surface receptors, help solubilize hydrophobic lipids, enhance the lipid uptake capacity of cells, and serve as enzyme cofactors in the metabolism of lipoproteins (18).

Different apolipoproteins constitute different lipoproteins, which influence their function. Apolipoprotein A-I, a 243–amino acid protein, is the major apolipoprotein component of HDL-C, which functions as an acceptor of cell membrane–free cholesterol in the reverse cholesterol transport pathway (19). apoA-II is the second most abundant protein component of HDL-C molecular. Protein and mRNA expression levels of apoA-I in the neuroretina and retinal pigment epitheliums were found to be significantly higher in diabetic donor eyes than in the non-diabetic donor eyes, thus suggesting that the protective mechanism within the retina acted through apoA-I by inhibiting lipid deposition and inflammation leading to DR (20). apoA-I has anti-inflammatory and anti-thrombosis properties. It also inhibits LDL oxidation, reduces vascular smooth muscle and endothelial cell damage, and helps to inhibit atherosclerosis (21). The mechanism of the protective effect functions in a way that apoA-I and apoA-II increase β cell insulin secretion and reduce the plasma level of glucose (22). In this study, both apoA1 and apoA-II resulted as protectors of DR and were negatively correlated with AI, fasting glucose, Glycated HbA1c, and API, which is consistent with previous study results (20, 21, 23). Interestingly, although little is known about the function of apoA-II, our results from multivariable logistic analysis strongly suggested that apoA-II acted as a protector in the pathogenesis of DR, which provides a new insight for finding new molecular targets for DR.

ApoB is a primary organizing component protein of LDL, very-low-density lipoprotein (VLDL), intermediate-density lipoprotein (IDL), and chylomicrons (CMs). ApoB has a significant role in lipoprotein transport (17, 24). apoB 100 is a major component of VLDL, LDL, and CMs; apoB 48 is a truncated form and a major component of CM. ApoB 48 is known as the only specific marker of intestinal CM particles (25). Previous *in vivo* and *in vitro* studies have indicated that apoB contributes to the development of DR and cardiovascular diseases (26, 27). On the other hand, plasma apoB was found to be significantly decreased following insulin analog initiation therapy (28). In this study, we further investigated the baseline characteristics of these enrolled patients, finding that about 33% of patients in DM, 53.7% of patients in NPDR, and 64.1% of patients in PDR groups were under insulin therapy. The higher proportion of insulin users in the NPDR and PDR groups may explain why apoB was not significantly correlated with the pathogenesis of DR in the present study, considering the decreased plasma level of apoB due to insulin therapy. Furthermore, the serum level of apoB could represent the atherosclerotic lipoprotein particles because each apoB can only bind one VLDL, LDL-C, or LPA. Non-LDL-C includes all atherosclerotic cholesterol in lipoproteins. Higher level of apoB/non–HDL-C means that there are more cholesterol-deplete atherosclerotic lipoprotein particles. The INTERHEART study had proved that lower apoB/non–HDL-C was correlated with lower risk of cardiovascular disease, which is consistent with our result (29).

ApoC-II (79 amino acids) and apoC-III (79 amino acids) are a major component of CMs and VLDL, which participate in the metabolism of these lipoprotein particles. At a lower level, the function of apoC-II binds to lipoprotein lipase (LPL) and, in turn, activates LPL after binding the surface of TG-rich lipoproteins. However, high serum level of apoC-II inhibits LPL activity (30). ApoC-III inhibits TG-rich lipoprotein lipolysis by reducing the LPL function and hepatic uptake of TG-rich lipoproteins. apoC-III has an important role in regulating the metabolism of triglyceride-rich lipoproteins (TRLs) (31). apoC-II and apoC-III have been identified as independent risk factors for hypertriglyceridemia (32, 33), cardiovascular disease, and T2DM (34, 35). In this study, we also found that the apoC-II/C-III ratio is associate with the occurrence and severity of DR. The mechanism behind may be due to the imbalanced serum level of apoC-II, and apoC-III contributes to the inhibition of LPL, resulting in excessive lipoproteins accumulation.

ApoE is a 299–amino acid plasma amphipathic alpha-helices and a major component of ligand for LDL receptor (LDLR) and LRP, CM remnants, VLDL, IDL, and some HDL-C. ApoE affects the LDLR, which is essential in the catabolism processing of triglyceride-rich lipoproteins. ApoE knockout mice were found to present high plasma cholesterol levels, which have been associated with the development of atherosclerosis (36) and brain–blood barrier breakdown (37). Subtypes of apoE have been implicated in Alzheimer’s disease, cardiovascular disease, and T2DM (38, 39). A previous study has shown that apoE gene polymorphisms are not associated with DR (40). In this study, we found that apoE was a risk for DR (OR = 1.30, *p* < 0.001); however, this result was not further confirmed by multiple variable logistic analysis (OR = 1.02, *p* = 0.848). ApoE/apoC-II resulted as a strong independent risk factor for the occurrence and severity of DR. Consequently, its subtypes (2, 3, 4) in the pathogenesis of DR should be further investigated (41).

LPA is a heritable, independent, and causal risk factor for atherosclerotic cardiovascular disease. In this study, we found that LPA was an independent risk factor for DR and further confirmed that the pathogenesis of DR compromised genetic factors, thus providing a future direction to further elucidate the genetic mechanisms of the occurrence and development of DR.

The main limitation of this study is that this was a case-control study and some of the confounder could not be eliminated; furthermore, the causal relationship between apos and severity of DR cannot be provided, and well-designed large cohort studies are warranted to replicate reported findings further. ACCORD and FIELD studies have found that fenofibrate can effectively slow down the progression of DR. Nonetheless, it remains unclear whether altering lipids and apolipoproteins can effectively prevent the onset or progression of DR, which should also be addressed by future long-term cohort studies. Furthermore, as apolipoproteins are components of different lipoproteins and can be defined as non-exchangeable or exchangeable proteins (such as apoA1, E, D, J, H, and M), it would have been more informative to analyze their levels separately in isolated lipoprotein classes to get casual aspects for the results presented in this study. In the coming study, we will isolate and characterize apolipoproteins to direct it to the therapeutic context.

In summary, this intriguing investigation revealed that the apolipoprotein ratios of apoC-II/apoC-III, apoE/apoC-II, and apoB/non–HDL-C were correlated with the presence of DM and DR. Furthermore, this ratio correlated with the retinopathy severity and resulted as a better predictor of DR than other traditional lipid measures. Our results raise the question of plasma apolipoprotein profile measurement that is necessary and can better predict the clinical outcome of patients with T2DM. We also found that apoA-I and apoA-II were protective factors for the occurrence and severity of DR. This study provides potential prevention biomarkers and therapeutic targets for DR.

## Data Availability Statement

The datasets presented in this article are not publicly available due to privacy or ethical restrictions. Requests to access the data that support the findings of this study should be directed to the corresponding author.

## Ethics Statement

The studies involving human participants were reviewed and approved by The Ethics Committee of Beijing Tongren Hospital, Capital Medical University. The patients/participants provided their written informed consent to participate in this study.

## Author Contributions

XZ contributed to conception and design of the study, drafted and revised the manuscript and perform statistical analysis. YN organized the database, enrolled patients, performed statistical analysis and drafted the manuscript. ZG performed statistical analysis and revised the manuscript. MZ provided comments and revised the manuscript. BQ and QW helped to enroll subjects. All authors contributed to manuscript revision, read, and approved the submitted version.

## Funding

This work was supported by the National Natural Science Foundation of China (Grant 81570850 and 82070988) and the Ministry of Science and Technology Foundation of China (Grant 2016YFC1305604).

## Conflict of Interest

The authors declare that the research was conducted in the absence of any commercial or financial relationships that could be construed as a potential conflict of interest.

## Publisher’s Note

All claims expressed in this article are solely those of the authors and do not necessarily represent those of their affiliated organizations, or those of the publisher, the editors and the reviewers. Any product that may be evaluated in this article, or claim that may be made by its manufacturer, is not guaranteed or endorsed by the publisher.

## Abbreviations

ACCORD, Action to Control Cardiovascular Risk in Diabetes; ADA, American Diabetes Association; AI, Atherogenic index; AIP, Atherogenic index of plasma; API, Atherogenic plasma index; apo, Apolipoprotein; ATP-III, Adult Treatment Panel-III; BCVA, Best-corrected visual acuity; BMI, Body mass index; CMs, Chylomicrons; DCCT, Diabetes Control and Complications Trial; DR, Diabetic retinopathy; EDIC, Epidemiology of Diabetes Interventions and Complications; FIELD, Fenofibrate Intervention and Event Lowering in Diabetes; HbA1c, hemoglobin; HDL-C, High-density lipoprotein cholesterol; IDL, Intermediate-density lipoprotein; LDL-C, Low-density lipoprotein cholesterol; LPA, Lipoprotein(a); LPL, Lipoprotein lipase; NCEP, National Cholesterol Education Program; NPDR, Non-proliferative diabetic retinopathy; PDR, Proliferative diabetic retinopathy; TC, Total cholesterol; TG, Triglyceride; TRLs, Triglyceride-rich lipoproteins; VLDL, Very low-density lipoprotein.
